# Centrifugation Versus Centrifugation-Free Stool Processing: Can the Simple One-Step Method Reliably Diagnose Pediatric Pulmonary Tuberculosis Using Xpert MTB/RIF Ultra?

**DOI:** 10.3390/diagnostics16020338

**Published:** 2026-01-21

**Authors:** S. M. Mazidur Rahman, Senjuti Kabir, Sabrina Choudhury, Sohag Miah, Tanjina Rahman, Md. Jahid Hasan, Mohammad Khaja Mafij Uddin, Arifa Nazneen, Shahriar Ahmed, Aung Kya Jai Maug, Sayera Banu

**Affiliations:** Infectious Diseases Division, International Centre for Diarrhoeal Disease Research, Dhaka 1212, Bangladesh; smmazidur@icddrb.org (S.M.M.R.); senjuti.kabir@icddrb.org (S.K.); sabrina.choudhury@icddrb.org (S.C.); sohag.miah1@icddrb.org (S.M.); tanjina.rahman@icddrb.org (T.R.); jahid.hasan1@icddrb.org (M.J.H.); kmuddin@icddrb.org (M.K.M.U.); arifa.nazneen@icddrb.org (A.N.); shahriar.ahmed@icddrb.org (S.A.); aung.maug@icddrb.org (A.K.J.M.)

**Keywords:** pediatric tuberculosis, Xpert MTB/RIF Ultra, stool, processing method, diagnosis, Bangladesh

## Abstract

**Background/Objectives**: Stool-based GeneXpert testing has become a useful approach for diagnosing pediatric pulmonary tuberculosis (PTB). This study compared two stool-processing methods, centrifugation-based processing (CBP) and simple one-step (SOS), for detecting PTB in children using Xpert MTB/RIF Ultra (Ultra). **Methods**: Children with presumptive PTB were screened cross-sectionally, and stool samples were collected and tested with Ultra using the CBP method from March 2022 to December 2024 across seven divisions of Bangladesh. A subset of stool samples (*n* = 281) that tested positive (*n* = 191) and negative (*n* = 90) by the CBP method were re-tested again with the same sample by Ultra using the SOS method. The results of the Ultra with SOS-processed stool were compared with the CBP method to evaluate overall agreement and detection efficiency across different bacterial burdens. **Results**: The SOS method detected 97 of 191 CBP-positive samples, resulting in a positive percentage agreement of 50.8% (95% CI: 43.5–58.1). All 90 Ultra-negative stool were also negative by the SOS method, yielding a negative percentage agreement of 100% (95% CI: 96.0–100.0). Overall agreement between the methods was 66.6% (Kappa: 0.398). The SOS method detected 100% of high- (4/4) and medium- (7/7), 97.3% (36/37) of low-, and 83.3% (35/42) of very-low-bacterial-burden samples, but only 14.9% (15/101) of the trace-detected samples that were identified by the CBP method. **Conclusions**: Stool testing with Ultra using the SOS processing method missed a significant number of the most prevalent form of child TB—the ‘trace-detected’ category identified by the CBP method. For increased detection of childhood TB nationwide, the national program should prioritize the use of Ultra on stool samples processed by the CBP method.

## 1. Introduction

Tuberculosis (TB), caused by the *Mycobacterium tuberculosis* complex (MTBC), remains a persistent global health problem and one of the top killers due to infectious diseases worldwide [1]. In 2023, the World Health Organization (WHO) estimated 10.6 million new TB cases and 1.6 million deaths globally, highlighting the urgent need for more accessible, sensitive, and rapid diagnostic tools, especially in high-burden and resource-limited settings [1].

The diagnosis of TB in children is difficult because of the presence of non-specific symptoms, and many do not show any symptoms in the early stages of the illness [2]. Diagnostic tests which are commonly used such as the chest X-ray is not specific, and the tuberculin skin test often provides false positive results [3,4]. The challenge is even greater for younger children, who cannot produce sputum for testing with WHO-recommended molecular diagnostics [5,6,7]. Confirming TB bacteriologically using respiratory specimens is also difficult due to the paucibacillary nature of the disease in children [8,9]. Recently, stool testing has become a promising alternative approach for the diagnosis of pulmonary TB in children unable to expectorate sputum [10,11].

Recent improvements in PCR-based molecular diagnostic assays, particularly the development of the Xpert MTB/RIF Ultra (Ultra) cartridge of the GeneXpert assay (Cepheid, Sunnyvale, CA, USA), have led to the increased detection of TB [12]. The Ultra provides better sensitivity compared to the earlier Xpert MTB/RIF (Xpert), making it more suitable for diagnosing childhood TB using stool samples [9,11,13]. Since 2021, the WHO has recommended stool as a new specimen type for both Xpert and Ultra as the initial diagnostic test for the detection of TB in children with signs and symptoms of pulmonary TB [13,14]. Implementation evidence from Asian countries like Indonesia, Pakistan, Tajikistan, and Bangladesh suggested that routine stool testing with Ultra at healthcare facilities could be cost-effective and feasible for increasing detection and treatment initiation of children with pulmonary TB [15,16,17,18]. However, the diagnostic performance of Ultra using stool samples depends mainly on the sample processing technique. Centrifugation-based processing (CBP) methods have been shown to enhance the concentration of MTBC by removing inhibitors and stool debris, thereby increasing the likelihood of detection by Ultra [19]. A simple one-step (SOS) stool-processing method has been developed that does not require the centrifugation step, and thus offers operational simplicity in field settings; however, the method may compromise sensitivity [14,20]. A recent multi-country study assessed different centrifugation-free stool-processing methods for detecting pulmonary TB in children. Compared to the microbiologically confirmed TB on sputum as the reference standard, Ultra showed a higher sensitivity on stool using the SOS method (38.6%) than other centrifugation-free approaches, such as the stool-processing kit (36.9%) and the optimized sucrose flotation method (31.3%). These results suggest that the SOS approach could be deployed widely at healthcare facilities [21].

Conversely, a study conducted in Bangladesh using the well-established CBP method on stool demonstrated a sensitivity of 58.6% for Ultra compared to microbiologically confirmed TB from induced sputum [9]. Notably, 80% of children with a positive stool Ultra result were classified as ‘Trace-detected’, indicating very low bacillary load. More recently, we have shown that programmatic implementation of Ultra using stool has significantly improved childhood TB diagnosis in Bangladesh using CBP procedures [18]. During this nationwide implementation, a similarly high proportion (73.7%) of stool Ultra-positive cases were found to be trace-positive, further highlighting the presence of the paucibacillary nature of TB in children in high-TB-burden countries like Bangladesh. Currently, the National Tuberculosis Control Programme (NTP) is scaling up stool testing nationwide using the centrifugation-free SOS method. However, comparative data between the SOS and centrifugation-based methods in children, particularly in those with very low bacterial burden, remain limited. In the current study, we aimed to determine whether the SOS stool-processing method can reliably detect the majority of pulmonary TB in children by Ultra, especially those with low bacillary loads, compared with the CBP method.

## 2. Materials and Methods

### 2.1. Study Settings, Design, and Population

**Figure 1:**
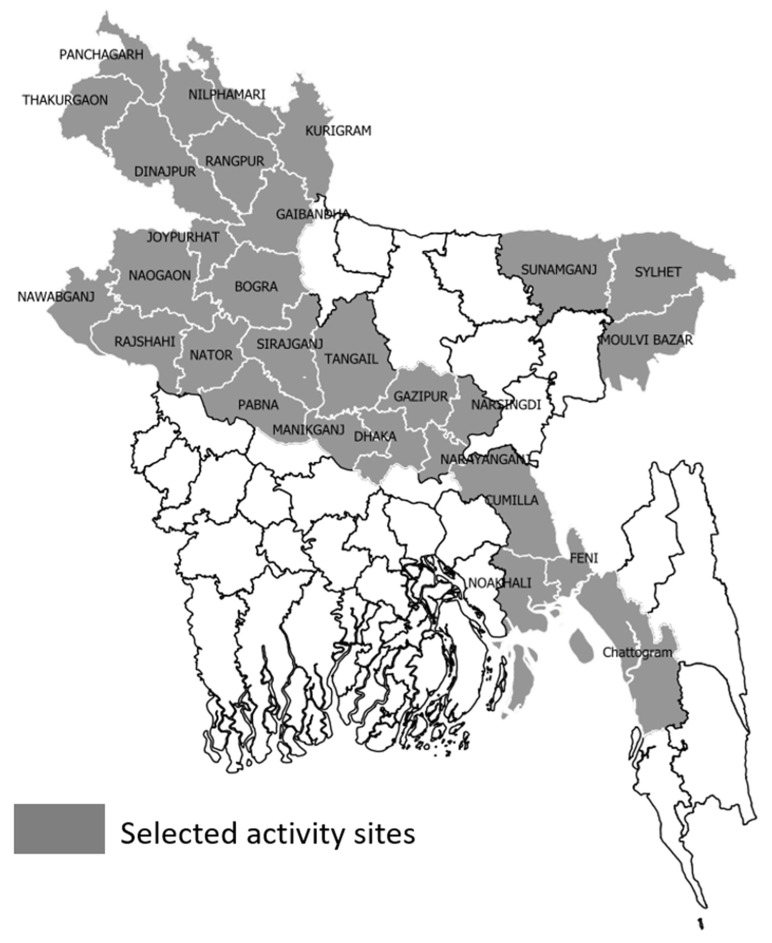
Geographic distribution of selected districts in Bangladesh where stool samples from children with presumptive pulmonary tuberculosis were tested using Xpert MTB/RIF Ultra. Shape file source: https://gadm.org/license.html, accessed on 24 December 2025.

**Figure 2:**
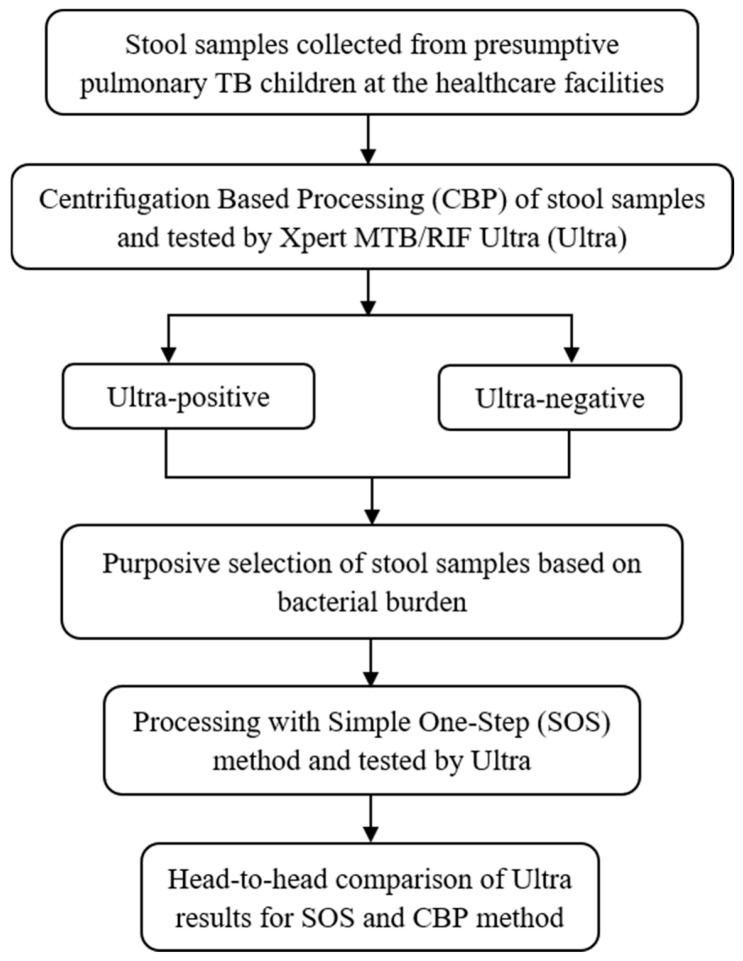
Flowchart of the overall study procedures from collection of stool samples from children with presumptive pulmonary tuberculosis to head-to-head comparison of the Xpert MTB/RIF Ultra results processed with the SOS and CBP methods.

In collaboration with the NTP, icddr,b had been implementing the USAID-funded Alliance for Combating TB in Bangladesh activity from March 2022 to December 2024. As part of this initiative, stool-based TB diagnosis in children was carried out at 51 healthcare facilities across 30 districts in five divisions of Bangladesh (Figure 1) [18]. A cross-sectional screening approach was adopted to identify presumptive pulmonary TB children. Children under 15 years of age were screened using a standardized screening toolkit with the criteria of cough ≥ 2 weeks, fever ≥ 2 weeks, weight loss, fatigue, and TB contact history within the past 12 months [18]. Those who screened positive with any of the criteria were evaluated by attending physicians at local hospitals and subsequently referred for Ultra testing using stool samples. This was a programmatic activity in which we facilitated only the routine TB diagnostic tests requested by the treating physicians. No additional diagnostic tests (such as mycobacterial culture) were performed, and no alternative respiratory specimens were collected from children for bacteriological confirmation. The identified presumptive pulmonary TB children underwent stool-based Ultra testing. Stool specimens were processed using a centrifugation-based concentration technique [9] at either nearby TB Screening and Treatment Centers (TBSTCs) operated by the International Centre for Diarrhoeal Diseases Research, Bangladesh (icddr,b) or transported to the central icddr,b Mycobacteriology Laboratory in Dhaka. Approximately 10–20 gm of stool (half of the container) was collected from each participant. To evaluate the SOS stool-processing method for detecting MTBC, we purposively selected a subset (*n* = 281) of stool samples with known results tested using the CBP method, and based on the availability of sufficient remnant samples to perform the SOS processing. This subset comprised 191 Ultra-positive samples, representing a range of bacterial loads, and 90 Ultra-negative samples. All selected samples were re-tested using the SOS method. The laboratory personnel who conducted the SOS-based stool processing were blinded to the results of Ultra performed on the CBP-processed samples. The overall study flowchart is shown in Figure 2.

The TB-diagnosed children were linked to the nearest NTP-affiliated directly observed treatment (DOT) centers for the initiation of anti-TB treatment. This study was approved by the Institutional Review Board (IRB) of icddr,b (Protocol number: PR24009). The activity we performed under programmatic implementation was part of the routine healthcare system, and the children did not undergo any additional procedures. Before screening, we explained our activity to the parents or caregivers accompanying the children visiting the healthcare facilities. We obtained verbal consent from them for collecting their socio-demographic and clinical information. We strictly maintained the confidentiality of respondents/participants and their health status and kept the data anonymous so that the respondents cannot be identified.

### 2.2. Stool Processing by the CBP Method and Testing with the Ultra Assay

Stool samples were processed according to the laboratory-optimized method, as described elsewhere [9]. Briefly, 2 gm of each stool specimen was aliquoted into a sterile 50 mL centrifuge tube. Next, 10 mL of sterile normal saline solution (0.9% NaCl) was added to the sample, and the mixture was homogenized thoroughly using a vortex mixer. Additional sterile normal saline solution (0.9% NaCl) was added to the tube until the total volume reached the 30 mL graduation mark. The homogenized stool suspension was then incubated at ambient temperature for 30 min. After incubation, 10 mL of the supernatant was carefully transferred to a new sterile 50 mL centrifuge tube and decontaminated with sodium hydroxide–sodium citrate solution (4% NaOH and 2.94% Na-citrate). The centrifuge tube was then filled with sterile phosphate-buffered saline (PBS, pH 6.8) up to the 40 mL mark. The suspension was centrifuged at 3000× *g* for 15 min. After centrifugation, the supernatant was discarded, and the resulting pellet was resuspended with 1.5 mL of PBS for subsequent Ultra testing, as per the manufacturer’s protocol. One mL of resuspended stool specimen was placed into a 15 mL centrifuge tube and mixed with sample reagent (SR) buffer at a 1:2 ratio. The sample buffer mixture was then incubated at room temperature for 15 min with manual agitation twice, and 2 mL was loaded into the Ultra cartridges. The cartridges were inserted into the GeneXpert platform, and the automated generated results were recorded.

### 2.3. Stool Processing by the SOS Method and Testing with Ultra Assay

Stool processing by the SOS method was followed as described in the WHO’s practical manual for the processing of stool samples for the diagnosis of childhood TB [14]. Briefly, a thumbnail-sized amount of stool was directly transferred from the stool container into the SR buffer bottle (containing 8 mL of buffer) using a wooden applicator. For liquid stool, 2.0 mL of SR buffer was discarded from the SR bottle before adding 2.0 mL of stool into the SR bottle. The mixture of stool and SR buffer was shaken vigorously for 30 s and then incubated at ambient temperature for 10 min. This step is repeated once. After incubation, the upper 2.0 mL of the suspension from the SR buffer bottle was carefully transferred into an Ultra cartridge. The cartridge was then loaded in the GeneXpert platform, and the automated generated results were recorded.

### 2.4. Statistical Analysis

Data was collected through structured electronic forms utilizing a web-based Android application. Screeners collected information on socio-demographics and other factors suggestive of presumptive child TB. The acquired data underwent routine quality assurance checks before being transferred directly to the central server. The analysis of data was conducted using the Stata statistical software (version 17.0; StataCorp LLC, College Station, TX, USA) and relevant statistical methods. Descriptive statistics (age, gender, and clinical symptoms) were summarized by frequencies and percentages. The performances of the CBP and SOS methods were compared based on overall Ultra positivity and positivity across different bacterial load categories. Chi-square or Fisher’s exact tests were applied, as appropriate, to compare proportions. The concordance of the results between the SOS and CBP methods was assessed by calculating the positive percentage agreement (PPA) and negative percentage agreement (NPA). The overall agreement between the methods was further evaluated using the Kappa coefficient [22]. Spearman’s correlation test was applied to calculate the correlation between the mean Ct values of the rpoB probes of the Ultra assay obtained from positive stool samples processed by CBP and their corresponding sample processed by the SOS method. For Ultra ‘Negative’ and ‘Trace’ detected categories, the Ct values for ropB probes were redefined as 41.0 (as the ropB probes do not amplify for ‘Negative’ and most of the ‘Trace’ categories). Statistically significant difference was considered at *p* < 0.05.

## 3. Results

### 3.1. Demographic and Clinical Characteristics

A total of 281 children were tested, with a median age of 6 years (interquartile range (IQR) 1.6–10.0). Of them, 86 (30.6%) were ‘≤2 years old’ and 86 (30.6%) were ‘5–9 years old’. Among symptoms, cough was reported by 241 (85.8%) participants, followed by fever (236, 84%), weight loss (220, 78.3%), and a history of TB contact (34, 12.1%) (Table 1).

### 3.2. Comparison Between the Centrifugation and SOS Methods for Detection of MTBC Using Ultra on Stool Samples

Among the 191 MTBC-positive stool samples identified by the centrifugation method, the SOS method detected 97 as positive, resulting in a PPA of 50.8% (95% confidence interval [CI]: 43.5–58.1). In contrast, all 90 stool samples that were negative by the centrifugation method were also negative by the SOS method, yielding a NPA of 100% (95% CI: 96.0–100.0). The overall agreement between the CBP and SOS methods was 66.6% (95% CI: 60.7–72.0) (Kappa: 0.398) (Table 2).

### 3.3. Comparison of the Centrifugation and SOS Methods Under Different Bacterial Burdens

**Figure 3:**
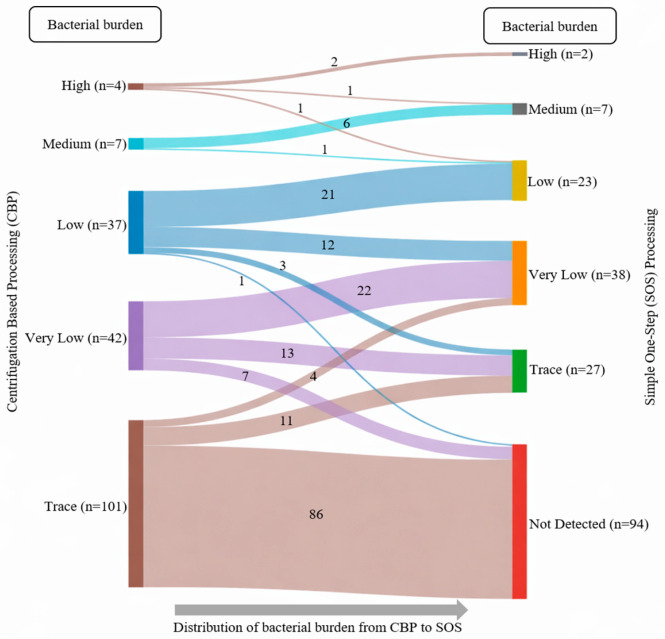
Sankey diagram illustrating the shifting of Xpert MTB/RIF Ultra’s bacterial burden between centrifugation-based processing (CBP) and the simple one-step (SOS) stool-processing methods. High (*n* = 4) and medium-burden (*n* = 7) categories identified by CBP were consistently detected by SOS, with minimal downgrading. Whereas, samples with low (*n* = 37), very low (n = 42), and trace-burden categories detected by CBP were frequently shifted to lower burden categories or not detected by SOS. Importantly, a majority of the trace category stool samples detected by CBP was not detected by SOS processing method (*n* = 86).

**Figure 4:**
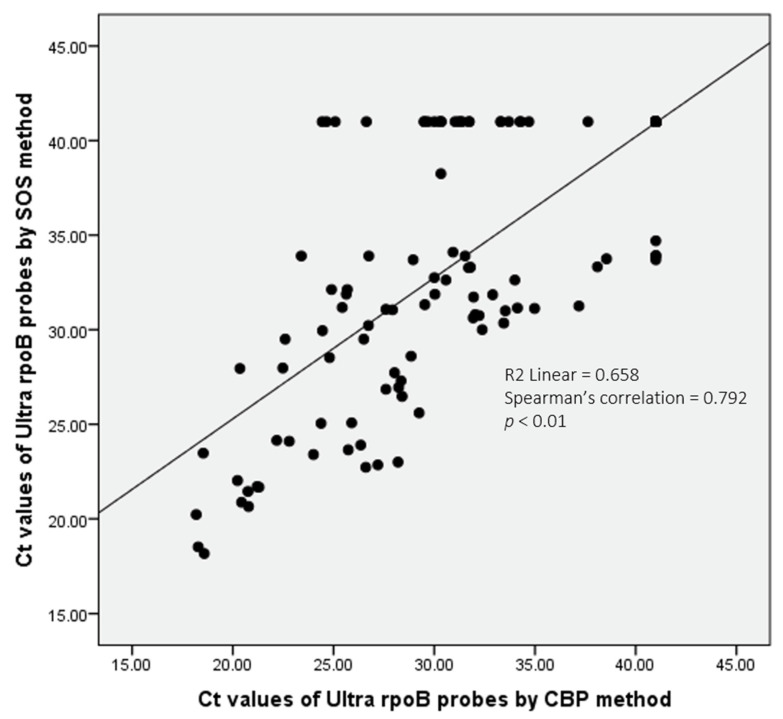
Correlation between the cycle threshold (Ct) values of the rpoB probes of the Xpert MTB/RIF Ultra (Ultra) assay obtained from stool samples processed by the centrifugation-based processing (CBP) and the simple one-step (SOS) methods. The Ct values of 191 positive stool samples processed by the CBP method showed a significant positive correlation with that of the SOS method. The best-fit line and Spearman’s regression R2 value are shown.

The comparative analysis of Ultra results using the CBP and SOS stool-processing methods revealed notable differences in MTBC detection, primarily influenced by bacterial burden (Table 3). The SOS method detected MTBC in all stool samples (*n* = 4) that showed a ‘High’ bacterial burden using the CBP method. Among these, two samples (50%) showed the same bacterial load with the SOS method, one (25%) downgraded to ‘Medium’, and another (25%) to ‘Low’. The SOS method also detected all stool samples (*n* = 7) with ‘Medium’ burden identified by the CBP method; six (85.7%) maintained the same bacterial load, while one (14.3%) was downgraded to ‘Low’. Among the samples with ‘Low’ bacterial burden (*n* = 37), 36 (97.3%) were detected by the SOS method. Of these, 21 (58.4%) remained as ‘Low’, 12 (33.3%) were downgraded to ‘Very Low’, and 3 (8.3%) to ‘Trace-detected’. For samples with ‘Very Low’ bacterial burden (*n* = 42) identified by the CBP method, 35 (83.3%) were identified by the SOS method. Among these, 22 (62.9%) remained ‘Very Low’ while 13 (37.1%) downgraded to ‘Trace-detected’. For the stool samples identified with the ‘Trace-detected’ category (*n* = 101) by the CBP method, only 15 (14.9%) were detected by the SOS method, which was significantly lower (*p* < 0.001) compared to the detection rate of SOS methods for other burden categories. Of these, 11 (73.3%) showed the same load, while four (26.7%) showed ‘Very Low’. The remaining 86 samples (85.1%) with ‘Trace-detected’ results by the CBP method were not detected by the SOS method (Table 3). A visual representation of the Ultra’s bacterial burden shifting from CBP to SOS processing methods is illustrated in Figure 3. The mean cycle threshold (Ct) values of the rpoB probes of the Ultra assay showed a significant positive correlation with the bacterial burden obtained from 191 positive stool samples processed by CBP and their corresponding sample processed by the SOS method (Spearman’s correlation = 0.792, *p* < 0.01) (Figure 4).

## 4. Discussion

Stool-based testing using the GeneXpert assay has gained recognition as a valuable tool for diagnosing pediatric pulmonary TB. Among the various processing techniques, CBP methods showed improved performance by effectively removing inhibitors and stool debris, thus enriching MTBC concentration for Ultra testing [19]. SOS and other centrifugation-free methods have been developed to streamline stool processing by eliminating the need for centrifugation, making it particularly suited for resource-limited and decentralized healthcare settings [14,20,23,24]. However, little is known about how bacterial burden influences the diagnostic performance of the methods, a critical gap that we intended to address in the current study. This study represents the first large-scale, head-to-head comparison of the SOS and CBP stool-processing methods to evaluate the performance of Ultra in detecting MTBC across a spectrum of bacillary loads under routine programmatic condition in Bangladesh.

When compared with the CBP method, the SOS method demonstrated a high level of specificity, with an NPA of 100%. This suggests that the SOS method is highly reliable in identifying the TB-negative samples correctly and thus has little chance of obtaining false positive results during field implementation. In contrast, the SOS method missed almost half of the positive cases identified by the CBP method, yielding a low PPA at 50.8%. The bacterial burden in the stool samples was closely linked to the variations in the detection performance. Compared to the CBP method, the detection rate of the SOS method was 100% in samples with ‘High’ and ‘Medium’ bacterial loads. Detection rates remained fairly high in ‘Low’ (97.3%) and ‘Very Low’ (83.3%) burden samples but reduced markedly for ‘Trace-detected’ samples (14.9%). A detailed comparison by burden between the two methods showed that the SOS method tends to lower the bacterial load compared to CBP. Only 50% of the stool samples that were categorized as having ‘High’ burden with CBP were also classified as such by the SOS method, and the remaining stool samples were downgraded to ‘Medium’ or ‘Low’ burden. This downgrade trend persisted across the ‘Medium’, ‘Low’, and ‘Very Low’ bacillary load groups, demonstrating that while SOS can detect MTBC, it often does not capture the full quantity of bacteria present in the stool for Ultra testing. This observation is further supported by the significant positive correlation in rpoB Ct values between the two processing methods, with SOS-processed samples consistently showing higher Ct values than those processed by CBP, reflecting reduced detectable bacterial load. In a recent experimental investigation, centrifugation-based processing outperformed the non-centrifugation approaches in detecting more positives at lower bacillary concentrations in spiked stool samples [19]. The fair agreement (kappa = 0.398) between the CBP and SOS methods, as demonstrated in our study, reflects the decreased capacity of the SOS method to detect MTBC, particularly in the lower-bacterial-burden stool samples such as in the ‘Trace-detected’ cases. This indicates that, programmatically, the SOS method should not replace CBP where centrifuge is available at healthcare settings for the diagnosis of pediatric TB.

One of the most critical aspects of pediatric TB is its paucibacillary nature, meaning that the bacterial load in clinical samples is often very low. This has been clearly demonstrated in our previous studies using Ultra on the centrifugation-based processing of stool samples, where approximately 70–80% of the positive cases fell into the ‘Trace-detected’ category, indicating a very low concentration of bacilli [9,18]. This consistently high proportion of trace-positive results, accounting for more than three-fourths of all positives, highlights the predominantly paucibacillary profile of childhood TB, not only in Bangladesh but also in other high-TB-burden countries [15,18,25,26]. Under such circumstances, the ability to detect these low-burden cases is pivotal. If only the SOS stool-processing method were used for Ultra, a substantial proportion, approximately 80%, of pediatric TB cases that could have been diagnosed microbiologically might be missed, resulting in the overall lower detection of child TB countrywide. In our recent programmatic implementation of stool Ultra activity, around 87% of ‘Trace-detected’ children identified via the CBP method were treated by the physicians, and most of these children demonstrated clinical improvement following treatment [18]. This indicates that the ’Trace-detected’ results represented the actual TB cases in children.

While the SOS method appears logistically and operationally simple, its lower performance, particularly for low-bacterial-burden cases, can result in missed or delayed diagnoses, leading to higher downstream costs related to repeat testing, inappropriate management, and increased morbidity and mortality in children. To balance cost-effectiveness and feasibility, NTP can take appropriate measures so that the stool samples from the lowest level of healthcare facilities could be transported to the designated centers equipped with centrifuges and skilled personnel such as secondary- and tertiary-level healthcare facilities, medical colleges, and specialized institutes. An alternative would be to establish a few primary-level healthcare clinics as regional processing hubs for neighboring locations. The NTP’s existing specimen transport network for MDR or rifampicin-resistant TB could be leveraged to support this approach, optimizing resource use while minimizing the risk and cost of misdiagnosis.

Although CBP demonstrated overall superior performance, the SOS method still has value under specific conditions. In our study, Ultra positivity with SOS was more frequently observed in samples with higher bacillary burden, suggesting that SOS is more likely to perform well in children with more extensive disease or higher mycobacterial load. Therefore, the SOS method may still be used in peripheral or primary-care settings where access to centrifugation is limited. In such contexts, a positive Ultra result obtained using the SOS method could support early treatment initiation, while negative results in clinically discordant children would require follow-up testing with Ultra using the CBP method at the designated centrifuge-equipped facilities through a clearly defined referral pathway. By employing such a tiered strategy, pediatric TB detection and treatment coverage may be increased, and Ultra cartridges could be used more efficiently.

Given the overall low positivity rate of Ultra on stool samples in Bangladesh, and the predominant nature of paucibacillary TB in children, we found a limited number of stool samples with high bacterial loads [9,18]. To ensure a balanced comparison, we purposively selected stool samples covering a range of bacterial load categories from CBP-positive samples collected across the country [18], which were used for comparison with the SOS processing method. This purposive selection may not fully represent the programmatic population but highlights the necessity of further evaluation at the programmatic level to adapt the best approach for the optimal detection of childhood TB in the country. As these samples were obtained through routine service delivery aimed at rapid TB diagnosis at the healthcare facilities, it was beyond our scope to perform microbiological tests (e.g., Ultra or culture) on respiratory specimens such as sputum/induced sputum or gastric aspirates. Consequently, a direct comparison of the CBP and SOS methods against the microbiological reference standard for the determination of sensitivity and specificity was not possible in this study. Future prospective studies are warranted to more comprehensively assess both the operational feasibility and diagnostic performance of the CBP and SOS processing methods, particularly for identifying low-burden childhood TB by Ultra under routine programmatic conditions.

## 5. Conclusions

The SOS stool-processing method showed a significantly reduced performance compared to the CBP method in detecting the most prevalent form of pediatric TB identified by Ultra—the ‘Trace-detected’ category, which typically represents low-burden disease. However, given its ability to detect samples with higher bacillary load, the SOS method may still be used, particularly in primary-care settings where access to centrifugation is limited. In such settings, negative SOS results in clinically discordant children should be referred for further confirmation using Ultra, following the CBP method. To enhance the detection of childhood TB with Ultra, the national program should take appropriate measures to prioritize the use of the CBP method across the country. This strategy will not only increase the detection of childhood TB but also ensure the cost-effective use of Ultra cartridges and other resources for optimum output.

## Figures and Tables

**Table 1:** Demographic and clinical characteristics of children with presumptive tuberculosis tested with Xpert MTB/RIF Ultra using stool samples.

Characteristics	Total (*n* = 281) (%)
Age in years	
Median (IQR)	6.0 (1.6–10.0)
≤2	86 (30.6%)
>2–<5	35 (12.5%)
5–9	86 (30.6%)
10–14	74 (26.3%)
Sex	
Male	152 (54.1%)
Female	129 (45.9%)
TB-suggestive symptoms	
Cough ≥ 2 weeks	241 (85.8%)
Fever ≥ 2 weeks	236 (84%)
Weight loss	220 (78.3%)
Fatigue	40 (14.2%)
TB contact history within past 12 months	34 (12.1%)

Note: IQR, interquartile range; TB, tuberculosis.

**Table 2:** Detection of *Mycobacterium tuberculosis* complex in stool samples of children by Xpert MTB/RIF Ultra using CBP and SOS processing methods.

Stool-Processing Method		CBP Method						
		Positive	Negative	Total	PPA (95% CI)	NPA (95% CI)	Overall Agreement (%)	Kappa
SOS method	Positive	97	0	97	50.8 (43.5–58.1)	100 (96.0–100)	66.6 (60.7–72.0)	0.398
	Negative	94	90	184				
	Total	191	90	281				

Note: CBP, centrifugation-based processing; SOS, simple one-step; PPA, positive percentage agreement; NPA, negative percentage agreement; CI, confidence interval.

**Table 3:** Comparison of bacterial loads detected by Xpert MTB/RIF Ultra in the stool samples of children following centrifugation and the SOS processing method.

Stool-Processing Method	Ultra-Negative (*n* = 90) *n* (%; 95% CI)	CBP Method				
		Ultra-Positive (*n* = 191)				
		High (*n* = 4) *n* (%; 95% CI)	Medium (*n* = 7) *n* (%; 95% CI)	Low (*n* = 37) *n* (%; 95% CI)	Very Low (*n* = 42) *n* (%; 95% CI)	Trace (*n* = 101) *n* (%; 95% CI)
SOS Method						
Negative	90 (100%; 96–100)	-	-	1 (2.70%; 0.1–14.2)	7 (16.7%; 7.0–31.4)	86 (85.1%; 76.7–91.4)
Positive	-	4 (100%; 39.8–100)	7 (100%; 59.0–100)	36 (97.3%; 85.8–99.9)	35 (83.3%; 68.6–93.0)	15 (14.9%; 8.6–23.3) *
Bacterial burden among detected samples						
High	-	2 (50%; 6.8–93.2)	-	-	-	-
Medium	-	1 (25%; 0.6–80.6)	6 (85.7%; 42.1–99.6)	-	-	-
Low	-	1 (25%; 0.6–80.6)	1 (14.3%; 0.4–57.9)	21 (58.4%; 40.8–74.5)	-	-
Very Low	-	-	-	12 (33.3%; 18.6–51.0)	22 (62.9%; 44.9–78.5)	4 (26.7%; 7.8–55.1)
Trace	-	-	-	3 (8.3%; 1.8–22.5)	13 (37.1%; 21.5–55.1)	11 (73.3%; 44.9–92.2)

Note: * Proportion test indicated significant difference (*p* < 0.001). CBP, centrifugation-based processing; SOS, simple one-step; Ultra, Xpert MTB/RIF Ultra; CI, confidence interval.

## Data Availability

The original contributions presented in this study are included in the article. Further inquiries can be directed to the corresponding author.
